# An iterative approach of protein function prediction

**DOI:** 10.1186/1471-2105-12-437

**Published:** 2011-11-10

**Authors:** Xiaoxiao Chi, Jingyu Hou

**Affiliations:** 1 School of Information Technology, Deakin University, Melbourne, Australia

## Abstract

**Background:**

Current approaches of predicting protein functions from a protein-protein interaction (PPI) dataset are based on an assumption that the available functions of the proteins (a.k.a. annotated proteins) will determine the functions of the proteins whose functions are unknown yet at the moment (a.k.a. un-annotated proteins). Therefore, the protein function prediction is a mono-directed and one-off procedure, i.e. from annotated proteins to un-annotated proteins. However, the interactions between proteins are mutual rather than static and mono-directed, although functions of some proteins are unknown for some reasons at present. That means when we use the similarity-based approach to predict functions of un-annotated proteins, the un-annotated proteins, once their functions are predicted, will affect the similarities between proteins, which in turn will affect the prediction results. In other words, the function prediction is a dynamic and mutual procedure. This dynamic feature of protein interactions, however, was not considered in the existing prediction algorithms.

**Results:**

In this paper, we propose a new prediction approach that predicts protein functions iteratively. This iterative approach incorporates the dynamic and mutual features of PPI interactions, as well as the local and global semantic influence of protein functions, into the prediction. To guarantee predicting functions iteratively, we propose a new protein similarity from protein functions. We adapt new evaluation metrics to evaluate the prediction quality of our algorithm and other similar algorithms. Experiments on real PPI datasets were conducted to evaluate the effectiveness of the proposed approach in predicting unknown protein functions.

**Conclusions:**

The iterative approach is more likely to reflect the real biological nature between proteins when predicting functions. A proper definition of protein similarity from protein functions is the key to predicting functions iteratively. The evaluation results demonstrated that in most cases, the iterative approach outperformed non-iterative ones with higher prediction quality in terms of prediction precision, recall and F-value.

## Background

Assigning biological functions to uncharacterized/un-annotated proteins is one of the major challenges in post-genomics due to the importance of proteins in various biological processes and the high cost of biological experiments [1]. On the other hand, new technologies in biology have generated various high-throughput protein-protein interaction (PPI) datasets. Meanwhile, function annotation schemes which give functional descriptions/definitions of protein functions also have been well developed, such as the Function Catalogue (FunCat) [2] and the Gene Ontology (GO) [3]. The research in protein interactions in living cells [4] shows that proteins interact with each other, rather than working alone, to perform their functions in various biological processes. Therefore, with the available protein interaction datasets and function annotation schemes, it is possible and feasible to use computational methods to predict functions for un-annotated proteins from protein interactions [5].

The past decade has seen a rapid development of computational methods for predicting protein functions from PPI datasets. To predict functions computationally, protein interactions in a PPI dataset are usually modelled as an undirected acyclic network. The nodes in the network represent unique proteins and the edges represent the interactions between proteins [6]. With this network model of protein interactions, various approaches have been proposed to predict functions of un-annotated proteins from the available information in the network and other related resources such as gene microarray profiles and the GO. In this paper, we focus our discussion on the prediction methods that are based on the protein and protein function similarities. The early Neighbour Counting method proposed by Schwikowski et al. [7] annotated an un-annotated protein with the functions that occurred most frequently among its neighbour proteins. This method could be considered as a simple similarity-based prediction method as it simply assigned similarity 1 (100%) to two proteins that have an interaction, or 0 if these two proteins have no interaction. Therefore the function scores that were used to predict functions were based on the function frequencies in the neighbour. Hishigaki et al. [8] improved Schwikowski's method by using the Chi-Square statistics instead of frequency as a scoring function. Brun et al. [9] improved the neighbour counting method by using a measure in graph theory to assign weights to the edges of a PPI network, and then used the weights as the similarities when predicting functions. In this method, the similarity was not 1 or 0 only anymore, it was within the range [0,1] instead. Samanta et al. [10] intended to improve the protein similarity definition by using a new distance metric and clustering techniques to compute the distance between two proteins. Chua et al. [11] extended Brun's and Samanta's ideas by including indirect neighbour proteins when predicting functions of an un-annotated protein. In recent years, more and more research turned to predicting protein functions semantically by combining the inter-relationships of function annotation terms in a scheme such as GO with the topological structure information in the PPI network. The inter-relationships are usually represented as functional similarities between annotation terms in the annotation scheme. To predict protein functions semantically, various methods were proposed to calculate functional similarities between annotation terms [12]. For instance, Resink [13] used the concept of information content to calculate the semantic similarity between two GO terms. Jiang et al. [14] and Lin [15] improved Resink's method by scaling the similarity to a fixed range. With the protein and protein function similarities, some methods were proposed to incorporate these similarities into the prediction, such as the k-Nearest Neighbour (kNN) based methods in [16].

The current approaches can predict functions effectively to some extent for some but not all cases. In addition to the factors such as incompleteness and noisy data of the PPI datasets, whether a computational algorithm can more reasonably reflect the nature of protein interactions will determine the quality of prediction. In fact the existing approaches, whether they are semantic or not, are based on an assumption that the functions to be predicted for an un-annotated protein are determined by the functions of annotated proteins in the dataset. That means the prediction is mono-directed from annotated proteins to un-annotated ones, and once the functions of un-annotated proteins are predicted, the prediction is finished (i.e. a one-off procedure). This assumption, however, only reflects one aspect of protein interactions. As a matter of fact, in real biological processes, proteins have high mobility and have dynamic interplay that produces a framework which is ever-changing but overall stable [17]. Proteins exchange their biological signals and share functions in a dynamic, rather than a static and mono-directed, circumstance. In other words, this dynamic feature of protein interactions should be reflected in function prediction procedures. The existing approaches, unfortunately, do not incorporate this dynamic feature into prediction procedures, and therefore do not validate whether the interactions between annotated and un-annotated proteins have achieved a stable state after the prediction is made.

Considering the above issues, in this paper, we propose an innovative approach to predict protein functions iteratively. The iterative prediction method simulates the dynamic process of protein interactions in terms of protein and function similarities when predicting functions. Meanwhile, in our algorithm the local and global semantic influence of the available protein functions in the dataset is also taken into account, which more reasonably counts the contribution of available functions to the prediction results. The iterative prediction starts with assigning initial predicted functions to the un-annotated protein, and then calculates the initial similarities between the un-annotated protein and its neighbour proteins. With these initial similarities, a kNN-based prediction method is applied to get the new predicted functions for the un-annotated protein. Replacing the initial/old predicted functions of the un-annotated protein, the new predicted functions are then used to recalculate the similarities between the un-annotated protein and its neighbour proteins for the next round of prediction. This prediction process is repeated until the similarities between the un-annotated protein and its neighbour proteins reach a stable state, which represents a dynamic stable status among the protein interactions in terms of similarities. To guarantee the prediction being conducted iteratively, a similarity between proteins must be properly defined. This is also one of our contributions in this paper.

The paper is organized as follows. In Section "Methods", we present the iterative prediction algorithm in detail. In Section "Results", we provide the evaluation results of our algorithm and the comparison results with the methods that are similar to our method. We discuss the concerns and issues that are related to our algorithm in Section "Discussion". We finally conclude our work in Section "Conclusions" and discuss some future work about iterative approach improvement.

## Methods

The idea of our prediction algorithm is to iteratively count the contribution of the available functions in the neighbours of the un-annotated protein to the final determination of predicted functions. The contribution of a function to the prediction is primarily dependent on the number of neighbour proteins that have the function and the similarities between the un-annotated protein and these neighbour proteins. In our algorithm, we also consider the similarities between the functions in the neighbour, as well as the global and local influence of the functions, in the prediction. The details of this iterative prediction algorithm are presented as follows. It can be seen that the base of our algorithm is the definitions of protein similarity and protein function similarity. Therefore, we firstly define these similarities, and then give the prediction algorithm.

Suppose the un-annotated protein is *p*, we denote the neighbour proteins of *p *as a set *N*(*p*). The neighbour proteins of a protein *p *are those that have direct and/or indirect interactions with *p *in the PPI network. In this paper, we only select those proteins that have direct interactions with *p *as the neighbour proteins of *p*. We also denote the functions of a protein *p' *as a set *F*(*p'*), and the functions of all the neighbour proteins of *p *as another set $FN\left(p\right)=\cup_{p^{\prime }\in N\left(p\right)}F\left(p^{\prime }\right)$. We use the GO terms [3] to annotate all the protein functions in our work.

Now we give the definitions of protein similarity and protein function similarity. For any two proteins *p *and *p'*, suppose the size of the set *F*(*p*) is *m *(i.e. the number of functions in *F*(*p*)), and the size of the set *F*(*p'*) is *n*. The similarity between two proteins *p *and *p' *is defined as

(1) $$sim\left(p,p^{\prime }\right)=\frac{1}{\max\left(m,n\right)}\sum_{f\in F\left(p\right)}\sum_{f^{\prime }\in F\left(p^{\prime }\right)}\delta_{f,f^{\prime }}$$

where *δ*_*f,f' *_is an indicator function, i.e. if *f *and *f' *are the same, its value is 1, otherwise, it is 0.

For any two functions *f *and *f'*, they can be represented as two vectors $\vec{f}$ and ${\vec{f}}^{\prime }$ whose element values indicate the occurrences of the GO notation terms that annotate the functions. If the number of terms/notations in GO is *t*, the dimension of each function vector $\vec{f}$ is then *t*. Since the GO is represented as a directed acyclic graph in which a GO term may have multiple parent GO terms, we call all parent terms of a GO term the ancestors of the term. If a function is annotated by a GO term, it is also annotated by the ancestors of the GO term. Therefore, the vector element values at the index positions that correspond to these ancestors are set to 1, otherwise set to 0. For example, suppose we have five GO terms for functional annotation (just for demonstration only), the function *f *is annotated by the fourth term whose ancestors are the second and third terms, and another function *f' *is annotated by the fifth term whose ancestors are the third and fourth terms, then these two functions *f *and *f' *can be represented as two vectors $\vec{f}=\left(0,1,1,1,0\right)$ and ${\vec{f}}^{\prime }=\left(0,0,1,1,1\right)$ respectively.

The similarity between two functions *f *and *f' *is then defined as

(2) $$fsim\;\left(f,f^{\prime }\right)=\vec{f}\cdot {\vec{f}}^{\prime }∕||\vec{f}||\cdot ||{\vec{f}}^{\prime }||$$

where $\vec{f}\cdot {\vec{f}}^{\prime }$ is the dot production of two vectors and $\left||\vec{f}|\right|$ is the norm of the vector $\vec{f}$. It can be seen from the above definition that the similarity between two functions is within the range 0 ≤ *f sim *(*f,f'*) ≤ 1. For the above two function vectors $\vec{f}=\left(0,1,1,1,0\right)$ and ${\vec{f}}^{\prime }=\left(0,0,1,1,1\right)$ for instance, $\vec{f}\cdot {\vec{f}}^{\prime }=2,\;||\vec{f}||=||{\vec{f}}^{\prime }||=\sqrt{3}$ and the similarity between these two function is *f sim *(*f,f'*) = 2/3.

With the above protein and protein function similarities, the score of the un-annotated protein *p *being annotated by a function *f *∈ *FN*(*p*), i.e. the contribution of function *f *to the final prediction results, is defined as:

(3) $$score\left(p,f\right)=\sum_{p^{\prime }\in N\left(p\right)}\left[sim\left(p,p^{\prime }\right)\times \left(\sum_{f^{\prime }\in F\left(p^{\prime }\right)}f\;sim\left(f,f^{\prime }\right)\times \log\frac{N}{n_{f^{\prime }}}\right)\right]$$

where *N *is the number of all proteins in the dataset and *n*_*f' *_is the number of proteins in the dataset that have the function *f'*. It can be seen from the equation (3) that the value of *f sim *(*f,f'*) refers to the local impact of available functions within the local domain *N*(*p*) on the prediction results, while the value $\log\frac{N}{n_{f^{\prime }}}$ reflects the global impact of available functions on the prediction results. Intuitively, if a function *f' *is common to almost all proteins, i.e. almost all proteins in the dataset have the function *f'*, then the importance as well as influence of *f' *decreases, otherwise it will increase.

The iterative function prediction is conducted based on the equation (3). In fact, for each available function *f *∈ *FN*(*p*), its contribution to the final prediction results is calculated by the score defined in (3). Therefore, all the functions in *FN*(*p*) can be ordered by their scores from the highest to the lowest, and then the first *k *functions with the *k *highest scores are selected as the predicted functions of the un-annotated protein *p*. The value of *k *is determined empirically or by the prediction requirements. In this paper, we select *k *as the average number of functions each protein has in the dataset. With the predicted functions of the un-annotated protein *p*, the similarities between the un-annotated protein *p *and its neighbour proteins, i.e. *sim *(*p,p'*) in (3), as well as the function scores are recalculated. With the recalculated scores, all the available functions in *FN*(*p*) are re-ordered and a new prediction is made. This procedure is repeated until the similarities between the un-annotated protein *p *and its neighbour proteins achieve a stable state.

To start the above iterative prediction procedure, we need to assign initial functions to the un-annotated protein *p*, so that the similarities between the un-annotated protein *p *and its neighbour proteins in (3) can be calculated. The selection of initial functions for the un-annotated protein *p *is determined by the initial function scores calculated by the equation (3) but with the similarity *sim *(*p,p'*) = 1 for any *p' *∈ *N*(*p*), i.e. for each function *f *∈ *FN*(*p*), its initial score is

(4) $$score^{\left(0\right)}\left(p,f\right)=\sum_{p^{\prime }\in N\left(p\right)}\sum_{p^{\prime }\in N\left(p^{\prime }\right)}\left[f\;sim\left(f,f^{\prime }\right)\times \log\frac{N}{n_{f^{\prime }}}\right]$$

We set the threshold for initial function selection as follows:

(5) $$\varepsilon =\frac{1}{size\left(FN\left(p\right)\right)}\sum_{f\in FN\left(p\right)}score^{\left(0\right)}\left(p,f\right)$$

where *size *(*FN *(*p*)) is the number of functions in the set *FN*(*p*). The functions whose scores calculated by (4) are over the threshold (5) are selected as the initial predicted functions of the un-annotated protein *p*.

It is observed from the above iterative prediction algorithm that the similarity definition of two proteins *sim *(*p,p'*) is the key to conducting the function prediction iteratively. If the protein similarity is defined in other ways rather than from protein functions, the prediction algorithm based on (3) is just a normal weighted kNN algorithm and the prediction cannot be conducted iteratively. So the prediction does not reflect the dynamic features of protein interactions, and it is just a one-off process. What makes our algorithm different from existing algorithms is that our protein similarity *sim *(*p,p'*) of two proteins *p,p' *is defined from their functions. With this similarity definition, the prediction algorithm based on (3) can go through an iterative process to predict functions until the similarities achieve a stable state. In other words, the prediction algorithm with our protein similarity definition reflects the dynamic features of protein interactions.

## Results

To evaluate the effectiveness of our iterative prediction algorithm, as well as to compare our method with other related methods, we used a real *S. Cerevisiae *protein-protein interaction (PPI) dataset derived from the BioGrid site (http://thebiogrid.org/) to build a protein interaction network for computational experiments. This dataset contained 232,239 interactions. To reduce the influence of noise data, we removed from the dataset the duplicated interactions, self interactions and all proteins that do not have GO annotation information. The filtered dataset for the experiments then contained 4,905 proteins, 3,260 GO terms and 155,662 interactions. The GO terms [3] and GO annotation dataset [18] used in the experiments were downloaded from http://www.geneontology.org/. We only used the biological process ontology and the GO annotations *of S. Cerevisiae *in our experiments.

Usually, the quality of a prediction algorithm is evaluated by its *precision, recall *and *F-value*, which are defined as follows:

(6) $$Precision=\frac{N_{P}}{N_{A}},\;Recall=\frac{N_{P}}{N_{R}},\;F-value=\frac{2\times Precision\times Recall}{Precision+Recall},$$

where *N*_*P *_is the number of correctly predicted functions for a given protein *p, N*_*A *_is the number of all predicted functions for protein *p, N*_*R *_is the number of real functions of protein *p*. For these evaluation metrics, *N*_*P *_is usually the number of predicted functions that exactly match the real functions. However, the function annotations of proteins have their specific features in the context of an annotation scheme such as the GO. It is known that the GO terms are organized in a hierarchical structure with the nodes representing the GO terms and the edges representing ancestor-child relationships. If a protein *p *is annotated by a node, it is also annotated by all ancestor nodes of that node. The ancestors of a node mean the more general function categories in biology. In other words, if two functions share some ancestors in the GO structure, even if they are not exactly the same, they are still similar to some extent at higher levels of functional categories. Therefore in our evaluations, in addition to evaluating how many functions we can predict that exactly match the real functions, we also evaluated to which extent the predicted functions are similar to the real functions over the function ancestor terms in GO. For this purpose, we adapted the evaluation method in [19] with our function similarity for algorithm evaluations. Actually, for a protein, suppose its real functional annotations are {*f*_*o*1_*, f*_*o*2_*, f*_o3_,..., *f*_*on*_}, and the predicted functional annotations are {*f*_*p*1_, *f*_*p*2_, *f*_*p*3_, ..., *f*_*pm*_}. The success of the prediction for a real function *f*_*oi *_(*i *= 1, ..., *n*) is defined as:

$$RecallSuccess\left(f_{oi}\right)=\max_{j}f\;sim\;\left(f_{oi},f_{pi}\right)$$

and the success of a predicted function *f*_*pj *_(*j *= 1, ..., *m*) is defined as:

$$PrecisionSuccess\left(f_{pj}\right)=\max_{i}f\;sim\left(f_{oi},f_{pj}\right)$$

The measures of new *recall *and *precision *are defined as follows:

(7) $$Recall=\frac{\sum_{i}RecallSucess\left(f_{oi}\right)}{\sum_{i}f\;sim\left(f_{oi},f_{oi}\right)},\;Precision=\frac{\sum_{j}PrecisionSucess\left(f_{pj}\right)}{\sum_{j}f\;sim\left(f_{pj},f_{pj}\right)}$$

The new *F-value *is defined as before but with the above new recall and precision definitions. These new recall and precision measures do make sense in biology because although two proteins interact with each other, they do not necessarily have the exact same functions, but they might be in more general function categories (i.e. the ancestors in GO). Therefore, the above new measures of recall and precision are more reasonable when assessing prediction quality in real biological applications.

Since our iterative algorithm is based on the cosine similarity between two function vectors, we named our algorithm the Cosine Iterative Algorithm (CIA). Due to the lack of existing similar iterative prediction algorithms, in our evaluation, we compared our CIA with two algorithms that were also based on the kNN method. One is Neighbour Counting (NC) [7] which predicted functions in the same way as the initial function prediction in our algorithm, but without iterations. Another one is the Iterative Neighbour Counting (INC) algorithm. The details of INC algorithm are as follows. We intended to evaluate whether the iterative approach (i.e. CIA and INC algorithms) produced better prediction results than the non-iterative approach (i.e. NC), and whether the cosine similarity based iterative algorithm (i.e. CIA) was better than the neighbour counting based iterative algorithm (INC).

With the INC algorithm, the score of a function *f *∈ *FN*(*p*) being assigned to the un-annotated protein *p *is calculated as follows:

$$score\left(p,f\right)=\sum_{p^{\prime }\in N\left(p\right)}\left[sim\left(p,p^{\prime }\right)\times I_{f,p^{\prime }}\right]$$

where the function *I*_*f,p' *_is defined as follow

$$I_{f,p^{\prime }}=\left\{\begin{array}{cc} 1 & f\in F\left(p^{\prime }\right)\\ 0 & f\notin F\left(p^{\prime }\right) \end{array}\right)$$

The initial score is calculated as follows:

$$score^{\left(0\right)}\left(p,f\right)=\sum_{p^{\prime }\in N\left(p\right)}I_{f,p^{\prime }}\;f\in FN\left(p\right).$$

The initial function selection for kicking off the iterative function prediction, as well as the iterative prediction procedure, is similar to our algorithm described above.

In our previous work [20], we have already compared the INC algorithm with another iterative algorithm that was based on Lin's similarity [15] of protein functions, i.e. the *I*_*f,p' *_in the above INC algorithm was replaced by the Lin's similarities between functions. Our previous evaluation results showed that the INC algorithm outperformed the Lin's similarity-based iteration algorithm in terms of precision, recall and F-value when predicting protein functions from different protein interaction datasets. Therefore in this paper, we focused on the comparison of CIA algorithm with the INC algorithm with respect to the iterative prediction quality.

**Figure 1:**
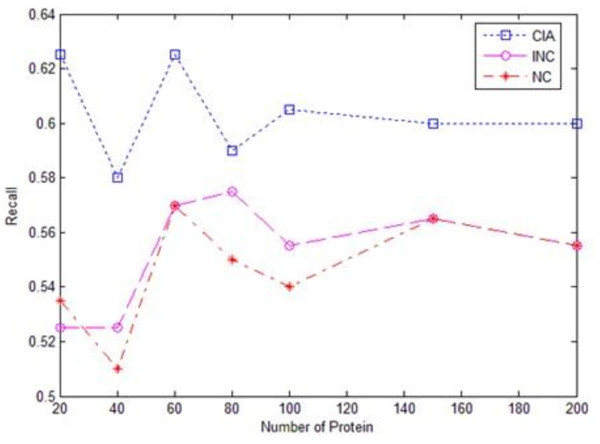
**Recall chart of three algorithms**.

**Figure 2:**
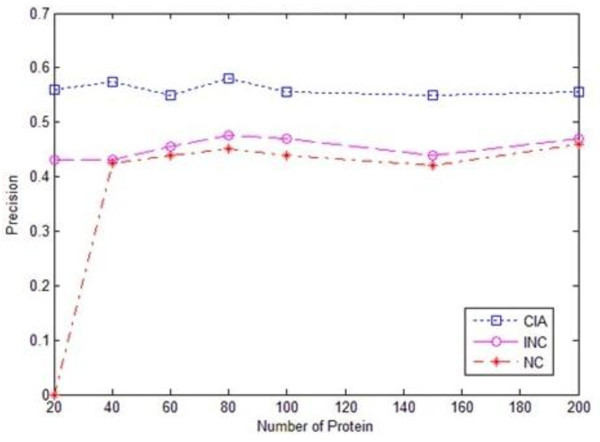
**Precision chart of three algorithms**.

**Figure 3:**
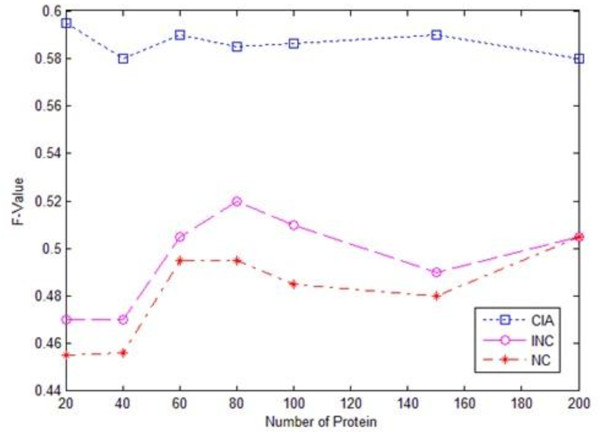
**F-value chart of three algorithms**.

Figures 1, 2 and 3 give the experimental results of the algorithms CIA, INC and NC regarding the recall, precision and F-value evaluations respectively. We chose five functions that had the first five highest scores as the prediction results in the evaluation, as the average number of functions each protein had in the dataset was around five. The evaluation was conducted on randomly selected test datasets with different sizes, ranging from 20 to 200. It was observed from the experimental results that iterative algorithms, CIA and INC, outperformed the non-iterative algorithm NC. For the iterative algorithms, the CIA algorithm performed better than the INC algorithm in terms of precision, recall and F-value. Meanwhile, the CIA algorithm was stable across the datasets, especially the large datasets.

**Figure 4:**
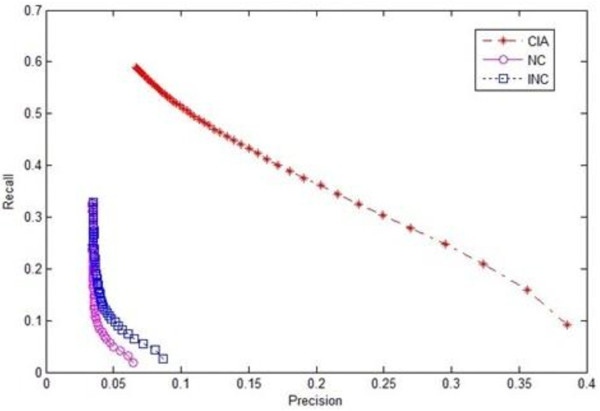
**Recall-Precision chart of three algorithms using original evaluation metrics in cross-validation experiments**.

**Figure 5:**
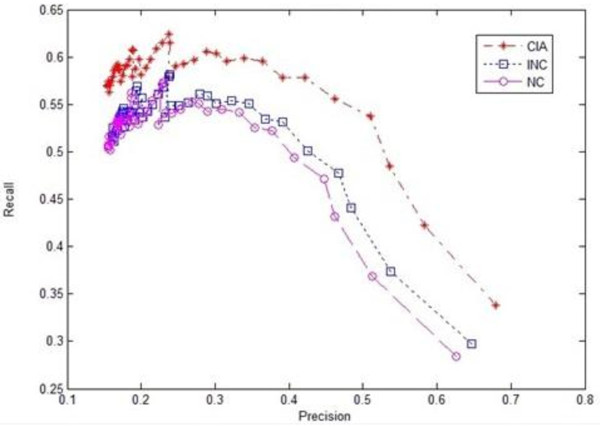
**Recall-Precision chart of three algorithms using new evaluation metrics in cross-validation experiments**.

To further evaluate the effectiveness of our algorithm, we conducted ten-fold cross-validation experiments. The original protein dataset was randomly divided into ten parts in the experiments. For each round of validation, one part was treated as a testing dataset and the remaining nine parts were treated as training datasets. The evaluation results for the three algorithms in terms of precision-recall are shown in Figures 4 and 5 respectively, where Figure 4 used the original definitions of precision and recall (6) and Figure 5 used the new definitions of precision and recall (7). The results demonstrated that the overall performance of our iterative prediction algorithm CIA was better than the other algorithms for both original and new definitions of precision and recall.

**Figure 6:**
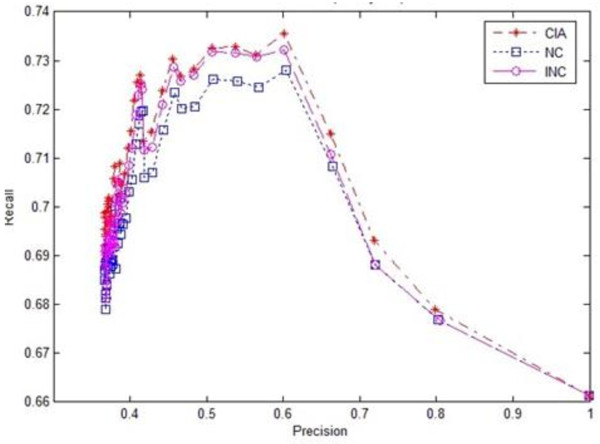
**Recall-Precision chart for Affinity-MS datasets**.

**Figure 7:**
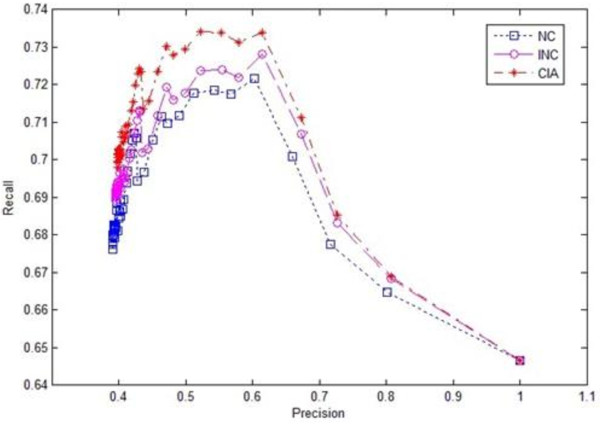
**Recall-Precision chart for Two-hybrid datasets**.

We also conducted cross-validation experiments on the protein interaction networks inferred by Affinity-MS and Two-Hybrid assays in the original BIOGRID database. The evaluation results for the three algorithms in terms of precision-recall are shown in Figures 6 and 7 respectively. The results also demonstrated that our algorithm CIA outperformed the other algorithms.

To demonstrate the effectiveness of our iterative approach, in Table 1 we provide some randomly selected sample prediction results from our iterative algorithm and the NC algorithm. It can be clearly seen from Table 1 that for most of the proteins, the iterative algorithm predicted an increasing number of correct functions when compared with the non-iterative algorithm.

**Table 1:** Sample prediction results

Protein	Real Function	NC	Iteration	GO description
YGR285C	GO:0006417	GO:0006417	GO:0006417	Regulation of translation
	GO:0006450	x	GO:0006450	Regulation of translational fidelity
	GO:0006457	x	x	Protein folding

YGL022W	GO:0006486	x	GO:0006486	protein amino acid glycosylation
	GO:0006487	GO:0006487	GO:0006487	protein amino acid N-linked glycosylation
	GO:0018193	GO:0018193	GO:0018193	peptidyl-amino acid modification
	GO:0009100	x	GO:0009100	glycoprotein metabolic process

YPR180W	GO:0006974	x	GO:0006974	response to DNA damage stimulus
	GO:0008152	x	x	metabolic process

YKL181W	GO:0006015	GO:0006015	GO:0006015	5-phosphoribose 1-diphosphate biosynthetic process
	GO:0009117	GO:0009117	GO:0009117	nucleotide metabolic process
	GO:0009156	GO:0009156	GO:0009156	ribonucleoside monophosphate biosynthetic process
	GO:0009165	GO:0009165	GO:0009165	nucleotide biosynthetic process
	GO:0031505	GO:0031505	GO:0031505	fungal-type cell wall organization
	GO:0043093	x	GO:0043093	cytokinesis by binary fission

YGL078C	GO:0006364	GO:0006364	GO:0006364	rRNA processing
	GO:0000027	x	GO:0000027	ribosomal large subunit assembly
	GO:0009451	x	x	RNA modification

YDR306C	GO:0006511	x	GO:0006511	ubiquitin-dependent protein catabolic process

YGR043C	GO:0005975	GO:0005975	GO:0005975	carbohydrate metabolic process
	GO:0008152	GO:0008152	GO:0008152	metabolic process
	GO:0006098	x	GO:0006098	pentose-phosphate shunt
	GO:0006914	x	x	Autophagy

YJR140C	GO:0006350	GO:0006350	GO:0006350	Transcription
	GO:0045449	GO:0045449	GO:0045449	regulation of transcription
	GO:0006368	x	GO:0006368	RNA elongation from RNA polymerase II promoter
	GO:0006336	x	x	DNA replication-independent nucleosome assembly
	GO:0000083	x	x	regulation of transcription involved in G1/S phase of mitotic cell cycle

YLR086W	GO:0007076	GO:0007076	GO:0007076	mitotic chromosome condensation
	GO:0007049	GO:0007049	GO:0007049	cell cycle
	GO:0007067	GO:0007067	GO:0007067	Mitosis
	GO:0051301	GO:0051301	GO:0051301	cell division
	GO:0000070	GO:0000070	GO:0000070	mitotic sister chromatid segregation
	GO:0030261	x	GO:0030261	chromosome condensation
	GO:0070058	x	x	tRNA gene clustering
	GO:0051276	x	x	chromosome organization

YJR065C	GO:0048308	GO:0048308	GO:0048308	organelle inheritance
	GO:0007015	GO:0007015	GO:0007015	actin filament organization
	GO:0000001	GO:0000001	GO:0000001	mitochondrion inheritance
	GO:0030833	x	GO:0030833	regulation of actin filament polymerization
	GO:0034314	x	x	Arp2/3 complex-mediated acting nucleation

**Note**: X stands for a not-predicted function. These sample results show that the iterative approach predicted more correct functions than the non-iterative approach. For instance, the NC algorithm predicted only one correct function for protein YGR285C, while the iterative algorithm predicted two correct functions; the NC algorithms failed in predicting any correct functions for protein YPR180W, however one function was correctly predicted by the iterative algorithm.

## Discussion

In this section, we discuss the concerns and issues related to our algorithm. The first concern is about the convergence of our iterative prediction algorithm. As stated in the section "Methods", the iterative prediction is based on iteratively updating the function scores calculated by equation (3). It can be seen from equation (3) that the score of a function *f *is determined by two factors, one is the influence of the function *f*, i.e. $\sum_{f^{\prime }\in F\left(p^{\prime }\right)}f\;sim\left(f,f^{\prime }\right)\times \log\frac{N}{n_{f^{\prime }}}$, another one is the similarity between the un-annotated protein *p *and its neighbour proteins, i.e. *sim *(*p,p'*). For a given function *f *∈ *FN*(*p*), its influence is fixed and will not be changed with the iterations. Therefore, the convergence of the algorithm depends on whether the similarities *sim *(*p,p'*), where *p' *∈ *N*(*p*), will be stable after finite iterations. In fact, according to equation (1), the similarity between two proteins depends on the functions they possess. Once their functions, especially the functions of the un-annotated protein *p*, are fixed, their similarity is fixed or stable. From the iterative algorithm, it can be seen that the final predicted functions should be those that have the highest influence and are highly similar to the functions of those proteins that are highly similar to the un-annotated protein *p*. However, those functions with an average influence but are highly similar to the functions of those proteins that are highly similar to the un-annotated protein *p*, or those functions with a higher influence but are on average similar to the functions of those proteins that are highly similar to the un-annotated protein *p*, are also the candidates of predicted functions. The initial function selection of the iteration algorithm only selects the most frequent functions that have the higher influence, without considering the impact of the protein similarity on the prediction results. After the first round of iteration, those functions are selected that have the highest influence and are highly similar to the functions of those proteins that are highly similar to the un-annotated protein *p*. As indicated above, since the neighbour of the protein *p *and the influence of the selected functions are fixed, the functions with the highest scores after the first round of iteration will still keep the highest scores in other iterations once they are assigned to the protein *p*, because the highest scored functions and protein similarities endorse each other in the iterations. Therefore, the second round of iteration is to select those candidate functions that have the second highest scores, and so on. That means the highest scores from the previous iteration will not be changed in the next iteration. Since the number of predicted functions is finite, after finite iterations (the number of iterations is less than or equal to the predefined number of predicted functions) the similarities between the un-annotated protein *p *and its neighbour proteins will not be changed any more, i.e. be stable. Therefore, the iterative algorithm is convergent. Our experiments also demonstrated that usually after two or three iterations, the predicted functions are stable.

Another concern about the iterative algorithm is whether the prediction results are sensitive to the initial function selection and the value of parameter *k *which determines the number of predicted functions. As analyzed above, the algorithm predicts functions by iteratively adjusting the similarities between the un-annotated protein and its neighbour proteins, and calculating the function scores. This iterative process has no specific constraints on the selection of initial functions, provided the candidate functions for iterations are selected as many as possible. Theoretically, we can select all available functions within the neighbour of the un-annotated protein as the initial functions for iterations. In our algorithm, we select initial functions according to their influence. This selection method is based on our prediction algorithm and an assumption that functions with higher influence are more likely to be the candidates of predicted functions. Therefore, this initial function selection method concentrates on those most likely candidate functions and reduces the computational cost. Our observation from the experiments demonstrated the effectiveness and efficiency of this initial function selection method, as we did not see significant differences between the prediction results produced from the method that selects all available functions in the neighbour as the initial functions and the prediction results produced from our initial function selection method. We believe that other existing prediction algorithms can also be used to select initial functions for our algorithm. Regarding the value of parameter *k *in the prediction, it is obvious that its value has impact on the prediction precision and recall, as well as the F-value. Ideally, this parameter value should be determined objectively. We tried to determine this value to be the number of functions whose scores were above the average score, or by ranking the function scores first and then determining the value of *k *to be the number of functions whose scores did not decrease sharply (e.g. less than 50%) between two adjacent functions in the ranking list. Our experiments showed that the current method of determining the value of *k*, i.e. the value of *k *is the average number of functions each protein has in the neighbour, achieved the best prediction results compared with other methods we tried. Whether there are better methods for determining the value of *k *is an issue we will address in the future research.

## Conclusions

This paper proposed a novel iterative approach trying to incorporate dynamic features of protein interactions into the protein function prediction. The iterative prediction algorithm also takes into account the local and global semantic influence of available functions within the protein interaction dataset on the prediction results. Therefore our approach is more likely to reflect the real biological nature between proteins when predicting functions. We adapted new evaluation metrics accordingly to evaluate the prediction quality of our algorithm and other similar algorithms. The evaluation results demonstrated that in most cases, the iterative approach outperformed non-iterative ones with higher prediction precisions and recalls. The prediction results also showed the feasibility and effectiveness of the proposed iterative approach. Since the iterations of the prediction algorithm occur within the neighbour of the un-annotated protein only, our iterative prediction algorithm can be scaled to other larger protein databases. It is concluded that the functions of an un-annotated proteins are mainly determined by the functions within the local domain (e.g. the neighbour) of the un-annotated protein, and those functions that are highly similar to all functions in the local domain and rare within the whole dataset are more likely to be the predicted functions of the un-annotated protein.

As we noticed, in our algorithm the prediction is based on the neighbour proteins of the un-annotated protein and their available information. In this paper, we only select those proteins that directly interact with the un-annotated protein as the neighbours. This neighbour selection method might lead to the genuine functions of the un-annotated protein being excluded from the final predicted functions. Further research is needed to select neighbours more reasonably to improve the prediction quality while reducing the impact of noise data. Another issue that comes to our notice is that in our algorithm we use a simple method to calculate the similarity of two proteins from their functions. Although this similarity calculation method significantly reduces the computational cost, it might not be able to precisely reflect the real similarity between proteins. Certain aggregation methods that make use of different data sources could be used to derive a more precise and reasonable protein similarity, and in turn, improve the prediction quality.

## Authors' contributions

XC participated in the study of prediction algorithms, carried out experiments and result analyses, and drafted the manuscript. JH conceived of and coordinated the study, carried out the study of prediction algorithms, participated in experimental result analyses, revised and finalized the manuscript. All authors read and approved the final manuscript.
